# HPV16 recruitment of SMARCAL1 to viral and host replication forks is required for the viral life cycle

**DOI:** 10.1128/mbio.00057-26

**Published:** 2026-03-30

**Authors:** Claire D. James, Aya H. Youssef, Jenny D. Roe, Floriana Cappiello, Francesca Antonella Aiello, Benedetta Perdichizzi, Rachel L. Lewis, Austin Witt, Apurva T. Prabhakar, Xu Wang, Molly L. Bristol, Pietro Pichierri, Iain M. Morgan

**Affiliations:** 1 Philips Institute for Oral Health Research, School of Dentistry, Virginia Commonwealth University (VCU) 6889, Richmond, Virginia, USA; 2 Department of Environment and Health Mechanisms, Biomarkers and Models Section, Istituto Superiore di Sanità 9289, Rome, Italy; 3 VCU Massey Comprehensive Cancer Center 172856, Richmond, Virginia, USA; Princeton University, Princeton, New Jersey, USA

**Keywords:** human papillomavirus, replication, life cycle, SMARCAL1, cervical cancer, head and neck cancer, therapy, homologous recombination

## Abstract

High-risk human papillomaviruses (HR HPVs) are responsible for around 5% of the world’s cancer burden. Activation and interaction with the host DNA damage response (DDR) promote the HPV16 life cycle. This study demonstrates a crucial interaction between HPV16 and SMARCAL1, a protein involved in the stabilization of stalled DNA replication forks. SMARCAL1 can complex with E2, is recruited to E1-E2 replicating DNA, and SMARCAL1 knockdown reduces the fidelity of E1-E2-mediated DNA replication in C33a cells but does not alter replication levels. SMARCAL1 is recruited to the HPV16 genome in HPV16-immortalized foreskin keratinocytes (HFK+HPV16), and *in situ* protein interaction with nascent DNA replication forks (SIRF) assays demonstrated that SMARCAL1 is hyper-recruited to host replication forks in HFK+HPV16 cells. Using COMET, DNA fiber, and cell growth assays, it was determined that knockdown of SMARCAL1 increased DNA damage and impaired replication fork progression in HFK+HPV16 cells, ultimately resulting in growth arrest. The viral genome integrates following SMARCAL1 knockdown in HFK+HPV16 cells. Therefore, SMARCAL1 facilitates host and viral DNA replication in HFK+HPV16 cells. Overall, the results demonstrate that HPV16 promotes SMARCAL1 recruitment to viral and host replication forks and that it is an essential factor for the HPV16 life cycle. The results expand our understanding of DDR proteins that regulate the HPV16 life cycle and suggest that inhibition of SMARCAL1 function represents a novel antiviral strategy for the treatment and prevention of HPV infections.

**IMPORTANCE:**

Human papillomavirus 16 (HPV16) is responsible for the majority of HPV+ cancers, contributing to 54% of cervical cancers and ~90% of HPV+HNSCC. Integration of viral genomes into host DNA can promote cervical cancer progression and correlates with poor prognosis in HPV-associated HNSCC, where around 70% of HPV+ cancers contain episomal viral genomes. Developing effective antiviral therapies requires a deeper understanding of the interplay between viral replication and host DNA damage response (DDR) pathways. This report demonstrates that SMARCAL1 is essential for HPV16 replication and keratinocyte proliferation and that its depletion leads to replication stress, DNA damage, and viral genome integration. This work underscores the delicate balance between viral exploitation of the host DDR and the risk of genome instability. These insights contribute to the broader understanding of HPV pathogenesis and may inform the development of therapeutic strategies targeting viral replication to prevent disease progression and improve clinical outcomes in HPV-associated cancers.

## INTRODUCTION

High-risk human papillomaviruses (HR HPVs) are etiologically linked to epithelial cancers, including ano-genital cancers and head and neck squamous cell carcinoma (HNSCC) (1). HPV is associated with around 80% of oropharyngeal cancers (HPV+OPC); the majority of patients with HPV+OPC respond better to radiotherapy (2–5). Previous work has shown that, whereas the viral genome is found to be integrated into host DNA in the majority of cervical cancers, HPV DNA is episomal in around 70% of HPV+HNSCC (6, 7). The virus requires the differentiation program of epithelia in order to complete its life cycle and utilizes host DNA damage response (DDR) proteins to replicate its genome (8, 9). In order to facilitate this, viral proteins E1 and E2 recruit host DDR factors to viral DNA; an active DDR is required for HPV life cycles (9–12). There are several components of the DDR pathway which facilitate the HPV life cycles; functional interactions occur between HPV16 and DDR factors including TOPBP1, SIRT1, WRN, BRD4, and SAMHD1, among others (13–19). Activation of the DDR by HPV16 promotes homologous recombination, which facilitates replication of the viral genome (20). During replication stress that occurs on the viral genome during the viral life cycle, fork reversal likely occurs, and several host factors maintain these forks and resolve the reversal to continue ongoing replication (21, 22). One factor required for fork reversal is SMARCAL1 (23, 24).

SMARCAL1 is a member of the highly conserved SNF2 ATP-dependent chromatin-remodeling enzyme family (23, 25, 26). This family functions in gene transcription, DNA damage repair, DNA recombination, DNA methylation, and cell cycle regulation. Interaction of SMARCAL1 with RPA, via the RPA32 subunit, at stalled replication forks recruits SMARCAL1 to sites of replication stress where it catalyzes the rewinding of single-stranded DNA (27–29). SMARCAL1 is recruited to adenovirus viral replication centers (30). Given the role of SMARCAL1 in fork reversal and previous involvement in DNA virus replication, we investigated the role of SMARCAL1 in the HPV16 life cycle.

Here, we demonstrate that SMARCAL1 is recruited to HPV16 E1-E2 transiently replicating DNA in the non-HPV cervical cancer cell line C33a. Removal of SMARCAL1 in C33a cells using shRNA does not alter the levels of E1-E2-mediated DNA replication, but does reduce the fidelity of replication. In HPV16-immortalized human foreskin keratinocytes (HFK+HPV16), SMARCAL1 is more recruited to host replication forks when compared with control cells. We recently demonstrated that E2 can induce the DDR during mitosis, and this could be contributory to DDR activation in the full HPV16 genome cells (31). SMARCAL1 is also recruited to the viral genome in HFK+HPV16 cells. Knockdown of SMARCAL1 in HFK+HPV16 attenuates cell growth; this does not occur in HFK expressing only the viral oncogenes E6 and E7. The HFK+HPV16 cells with SMARCAL1 knockdown have slower host replication fork progression, increased DNA damage, and integrated viral genomes. The results present a model in which the presence of HPV16 promotes recruitment of SMARCAL1 to viral and host DNA for correct viral and host replication fork progression. In the absence of SMARCAL1, the presence of HPV16 results in attenuation of host replication fork progression, increased double-stranded DNA breaks (likely due to replication fork collapse), and the abrogation of cellular growth. Overall, the results present SMARCAL1 function as a therapeutic target for disrupting the growth of HPV16-positive cells, including cancers that retain episomal viral genomes.

## RESULTS

### SMARCAL1 interacts with the viral replication complex and regulates viral replication fidelity

**Fig 1:**
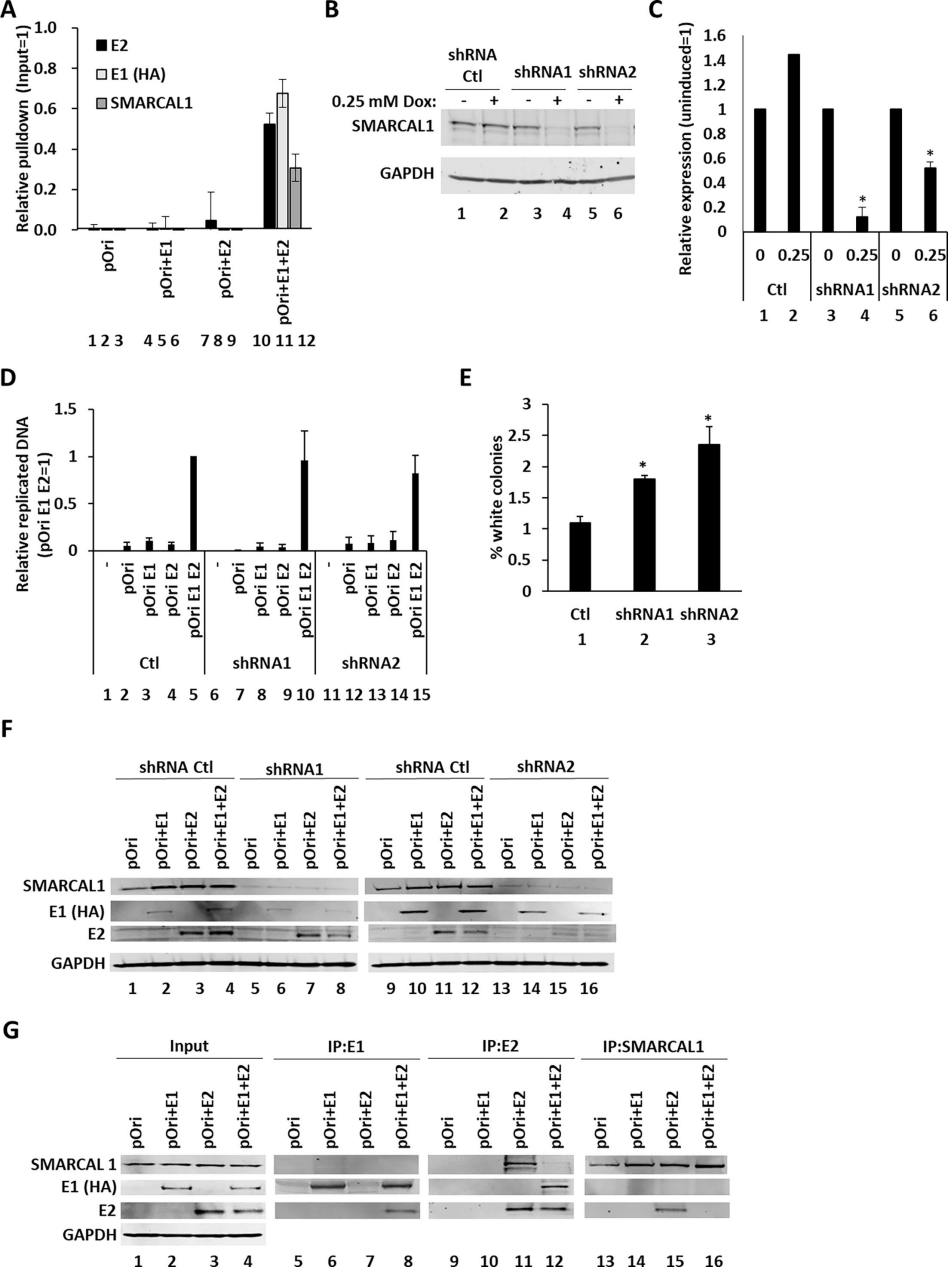
SMARCAL1 is recruited to E1-E2 replicating DNA. (**A**) C33a cells were transfected with 1 μg of pOri (a plasmid containing the HPV16 origin of replication), 1 μg of an HPV16 E1 expression vector (HA-tagged), and 1 μg of an HPV16 E2 expression vector. Forty-eight to 72 h following transfection, chromatin was prepared and ChIP assays carried out using E2, HA (for E1), and SMARCAL1 antibodies. The results are representative of a summary of three independent experiments. With all antibodies, there is a significant increase in pOri signal when E1 and E2 are expressed, versus no viral protein or either by itself. (**B**) C33a cells expressing doxycycline (Dox)-inducible control shRNA (shRNA Ctl) or two targeting SMARCAL1 (shRNA1 and shRNA2) were generated and 0.25 mM of Dox added to confirm SMARCAL1 knockdown with the targeting shRNAs. (**C**) This confirms SMARCAL1 RNA knockdown in the C33a cells represented in panel **B**. (**D**) C33a cells were transfected with 1 μg of pOri, 1 μg of an HA-E1 expression vector, and 1 μg of E2 expression vector. Forty-eight to 72 h following transfection, cells were harvested and processed for DNA replication determination as described in Materials and Methods. The results presented represent the summary of at least three independent experiments. (**E**) The DNA harvested in panel **D** was electroporated into bacteria, and the number of white colonies determined on agar-X-gal plates. The number of white colonies identifies unfaithful replication. (**F**) C33a cells in panels B and C were treated for 48 h with 0.25 mM Dox and transfected with 1 μg of pOri, 1 μg of an HA-E1 expression vector, and 1 μg of E2 expression vector. Protein extracts were prepared 48–72 h later, and western blotting was carried out as indicated. (**G**) C33a cells were transfected with 1 μg of pOri, 1 μg of an HA-E1 expression vector, and 1 μg of E2 expression vector, and proteins were extracted 48–72 h later. The indicated immunoprecipitations followed by western blotting are shown. *, *P*-value < 0.05.

The recruitment of DDR factors to HPV replication centers demonstrates replication stress occurring on the viral genome. Our prior work demonstrated that WRN is critical for viral replication, suggesting that the process of replication fork reversal is critical for viral replication (17). To investigate whether SMARCAL1, a host protein also involved in replication fork reversal, is recruited to E1-E2 replicating DNA, C33a cells were transiently transfected with plasmids containing the HPV16 origin of replication (pOri) and expressing the viral replication factors E1 and E2. Chromatin was isolated 48 h following transfection, and ChIP was performed using antibodies to detect SMARCAL1, E1 (HA, as the E1 expression vector is HA-tagged), and E2; all three factors were recruited to the E1-E2 replicating DNA (Fig. 1A, lanes 10–12). Lanes 1–3 were transfected only with the plasmid containing the HPV16 origin (pOri), lanes 4–6 with pOri plus an E1 expression vector, and lanes 7–9 with pOri plus an E2 expression vector. Our previous work has demonstrated both E1 and E2 expressions are required for viral factor recruitment, which in turn recruits host factors (32).

To assess the functional impact of SMARCAL1 recruitment to E1-E2 replicating DNA, we generated C33a cell lines with inducible shRNA targeting SMARCAL1. Western blot analysis confirmed the knockdown of SMARCAL1 protein expression with two separate shRNAs following incubation of cells with 0.25 μM doxycycline for 48 h (Fig. 1B, lanes 4 and 6). qRT-PCR also confirmed knockdown of SMARCAL1 RNA (Fig. 1C, lanes 4 and 6). Cell lines containing inducible control shRNA showed no decrease in SMARCAL1 protein or RNA following doxycycline treatment (Fig. 1B and C, compare lanes 1 and 2). Following induction of shRNA, cells were transfected with E1, E2 expression vectors, and pOri, and DNA harvested 48 h later and processed for measurement of E1-E2-mediated DNA replication levels (32). Knockdown of SMARCAL1 did not significantly alter E1-E2-mediated DNA replication (Fig. 1D, compare lanes 10 and 15 with lane 5). pOri contains the bacterial LacZ gene, and we have used this to determine replication fidelity. In this assay, the pOri plasmid is replicated by the co-expressed E1 and E2 proteins, and the replicated DNA is harvested from the cells. Input DNA is removed using DpnI, which does not cut mammalian replicated DNA as it is not modified (DpnI cuts at bacterially methylated target sites). The levels of replication are then determined using PCR, and the fidelity of replication is determined by transfecting the replicated DNA into bacteria and growing them on X-gal plates; the percentage of white colonies signals mutations in the LacZ gene introduced during E1-E2 replication (33). While the replication assays showed no significant change in levels following SMARCAL1 knockdown, mutation frequency increased significantly, indicating reduced viral replication fidelity in the absence of SMARCAL1 (Fig. 1E, compare lanes 2 and 3 with lane 1). Knockdown of SMARCAL1 did not significantly alter expression of the E1 and E2 proteins in these C33a experiments (Fig. 1F).

To investigate whether the viral replication factors could directly interact with SMARCAL1, immunoprecipitation (IP) experiments were performed (Fig. 1G). Input levels of proteins for the IPs are shown in lanes 1–4, E1 (HA) in lanes 5–8, E2 in lanes 9–12, and SMARCAL1 in lanes 13–16. E1 is unable to co-IP SMARCAL1 (lane 6) but, as expected, interacted with E2 (lane 8). E2 could co-IP SMARCAL1 when expressed by itself (lane 11) but was unable to do so when E1 is co-expressed (lane 12); E1 interacts with E2 as expected (lane 12). SMARCAL1 can interact with E2 (lane 15), but not when E1 is co-expressed (lane 16). This presents a model where E2 may be involved in recruiting SMARCAL1 to viral DNA, but following E1 complexing with E2, SMARCAL1 is removed from E2. This is presumably due to a high-affinity interaction between E1 and E2 displacing SMARCAL1 from E2. This mechanism would promote recruitment of SMARCAL1 to the viral genome. Alternatively, E2 interaction with SMARCAL1 could be independent from the role of SMARCAL1 in regulating viral replication. E2 regulates host gene transcription, and SMARCAL1 can regulate the structure of promoter regions to influence transcriptional regulation (34, 35). Therefore, the role of the E2-SMARCAL1 interaction may be in regulation of host gene transcription.

### SMARCAL1 is essential for HPV-driven cell growth

**Fig 2:**
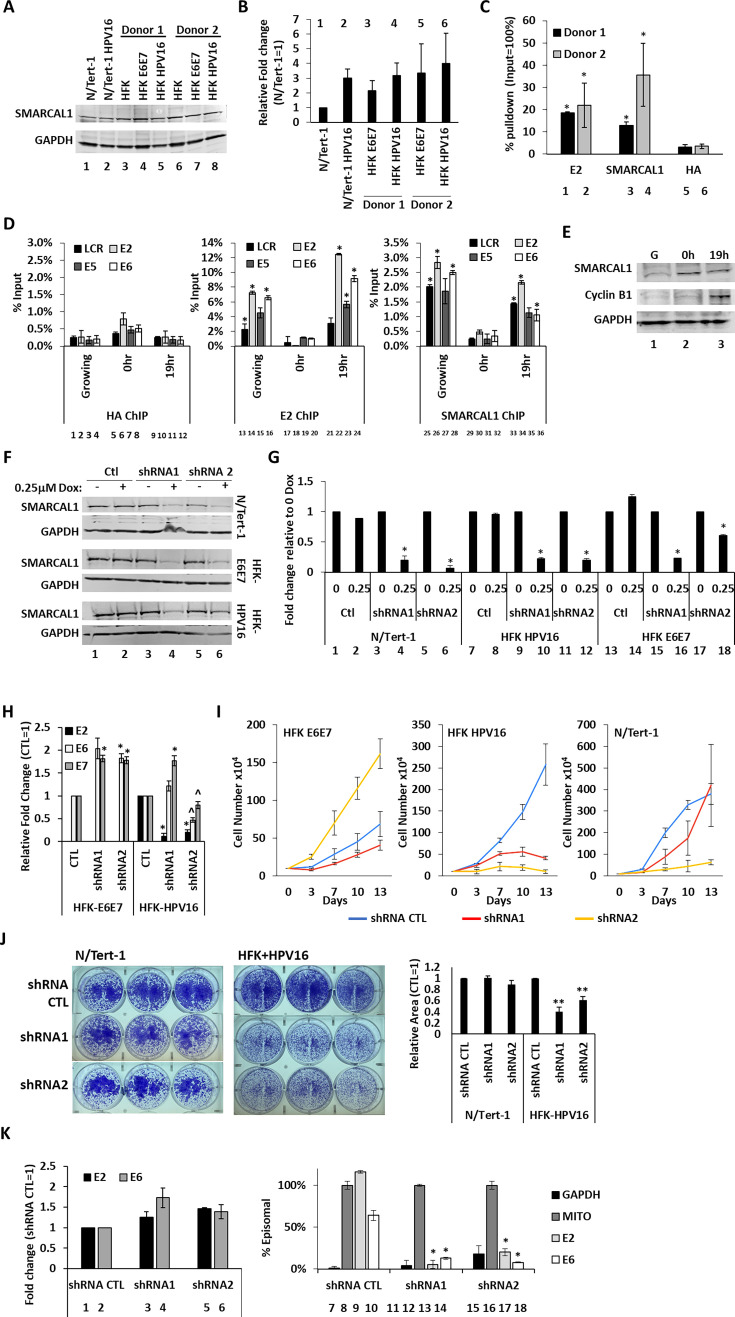
SMARCAL1 is critical for the growth of HFK+HPV16 cells. (**A**) Western blots demonstrate that SMARCAL1 levels are not substantially changed by the presence of HPV16. (**B**) SMARCAL1 RNA levels were not significantly increased by HPV16. (**C**) Chromatin was prepared from two HFK+HPV16 (human foreskin keratinocytes immortalized with HPV16) cell lines generated from two different donors and immunoprecipitated (ChIP) with E2, SMARCAL1, and HA (as a negative control) antibodies, and the presence of the viral origin detected using qPCR. The results represent the summary of at least two independent experiments. (**D**) HFK+HPV16 cells were double-thymidine blocked to generate G0/G1 cells (0 h) or released for 19 h which enriches for mitotic cells. (**E**) Western blotting for cyclin B1 demonstrates enrichment of mitotic cells following 19 h release from the double-thymidine block. (**F**) N/Tert-1+Vec (containing pcDNA as a control), HFK+E6E7 (immortalized with the viral oncogenes E6 and E7 only), and HFK+HPV16 were generated expressing inducible shRNA control (Ctl) or inducible shRNAs targeting SMARCAL1 (shRNA1 and shRNA2). The cells were treated with 0.25 mM Dox for 48 h prior to protein harvesting, and western blotting confirmed knockdown of SMARCAL1 in the appropriate samples. (**G**) Knockdown of SMARCAL1 RNA via the shRNA targeting sequences was confirmed. (**H**) RNA levels of E2, E6, and E7 were determined following SMARCAL1 knockdown. (**I**) Growth curves of the indicated cell lines were carried out over the period indicated, cells were trypsinized and passaged at each of the days indicated. The results shown represent the summary of three independent experiments. (**J**) The indicated cell lines were grown long-term following SMARCAL1 knockdown, and the results are quantitated to the right. (**K**) Viral genome status was investigated using TV exonuclease assays following knockdown of SMARCAL1 and the episomal status of the E2 and E6 signal determined relative to that of mitochondrial DNA, which, like the viral genome, is episomal. *, *P*-value < 0.05.

Figure 1 demonstrates the involvement of SMARCAL1 in the regulation of E1-E2-mediated DNA replication in C33a cells, prompting investigation of the role of SMARCAL1 in the HPV16 life cycle. Figure 2A demonstrates that the presence of the HPV16 genome or the viral oncogenes by themselves does not regulate SMARCAL1 protein levels. While there was some increase in SMARCAL1 RNA levels in the presence of HPV16 (Fig. 2B), the differences did not reach statistical significance. ChIP assays were carried out with chromatin isolated from keratinocytes immortalized with the full HPV16 genome (HFK+HPV16), utilizing SMARCAL1 antibodies, with HA and E2 antibodies as negative and positive controls (the E1 in the HPV16 genome is not tagged with HA, as it is in the C33a experiments carried out in Fig. 1), respectively. In two donor keratinocyte backgrounds, SMARCAL1 and E2 (Fig. 2C, lanes 1–4) were precipitated with viral DNA at significantly greater levels than the HA negative control antibody (lanes 5 and 6). To determine whether the E2-SMARCAL1 interactions were genome-wide and whether they were cell cycle regulated, we carried out further ChIP analysis. Donor 1 HFK cells were synchronized into G0/G1 using double thymidine blocking (DTB), a protocol we have used to cell cycle synchronize HFK+HPV16 cells (31). Releasing them from the block for 19 h enriches for cells in mitosis, and we have recently demonstrated that differentiating HFK+HPV16 cells, which support the viral life cycle, have a mitotic phenotype as evidenced by enhanced cyclin B1 expression (36). Figure 2D demonstrates that in DTB cells, there is no increased interaction of E2 or SMARCAL1 with any part of the viral genome when compared with the negative HA control antibody (compare lanes 17–20 and 29–32 with lanes 1–12). There is increased SMARCAL1 and E2 recruitment to the viral DNA in growing cells and also in mitotic cells (lanes 13–16 and 21–24 for SMARCAL1, respectively, and lanes 25–28 and 33–36 for E2, respectively). There were no consistent differences in SMARCAL1 or E2 distribution on the viral genome when comparing the LCR with different parts of the viral genome. These results demonstrate that SMARCAL1 and E2 engage with the viral genome only when replication is occurring, as they fail to bind in G0/G1-arrested cells. Western blotting confirmed enrichment of cyclin B1 at the 19 h time point (Fig. 2E, compare lane 3 with lanes 1 and 2).

To investigate the role of SMARCAL1 in the HPV16 life cycle, HFK+HPV16 lines were established expressing either vector control (Ctl) or doxycycline-inducible shRNAs targeting SMARCAL1 (shRNA1–3). This was done in HFKs immortalized with either HPV16 full genome (HFK+HPV16) or the oncoproteins E6E7 (HFK+E6E7), or telomerase (N/Tert-1); the HFK+HPV16 and HFK+E6E7 were isogenic. shRNA expression was induced by culture in 0.25 μM doxycycline, and western blotting confirmed reduced SMARCAL1 protein expression following shRNA targeting of SMARCAL1, when compared with control shRNA (Fig. 2F, compare lanes 4 and 6 with lane 2). qRT-PCR confirmed significant RNA knockdown of SMARCAL1 with the targeting shRNAs when compared with control shRNA (Fig. 2G). SMARCAL1 knockdown significantly reduced E2 RNA levels, presumably due to viral genome integration (see below), while the effects on E6 and E7 RNA levels were inconsistent, demonstrating no statistically significant reduction in expression of the viral oncogenes following SMARCAL1 knockdown. (Fig. 2H). To determine the effect of SMARCAL1 knockdown on cell growth, cells were counted over a 13 day period with passaging at days 3, 7, and 10 (Fig. 2G). There was a variable effect on cell growth in N/Tert-1 and HFK+E6E7 cells following SMARCAL1 knockdown (Fig. 2I, left and right panels). However, in HFK+HPV16 cells, SMARCAL1 knockdown ultimately resulted in a growth stop (Fig. 2I, middle panel). SMARCAL1 knockdown using both targeting shRNAs resulted in a significant reduction in growth of HFK+HPV16 cells. We carried out colony formation assays with N/Tert-1 and HFK+HPV16 and demonstrated a significant long-term attenuation of growth only in the HFK+HPV16 cells (Fig. 2J).

Given the recruitment of SMARCAL1 to replicating HPV16 DNA in HFK+HPV16 cells (Fig. 2C and D), and the role of SMARCAL1 in managing DNA replication stress, the genome status of HPV16 was determined following SMARCAL1 knockdown. The most quantitative assay for determining HPV16 genome status was recently demonstrated to be the TV exonuclease assay as it can measure the levels of both integrated and episomal genomes and was the most reflective of DNA-seq data (37). This approach was used to investigate the HPV16 genome status following SMARCAL1 knockdown. Following SMARCAL1 knockdown, there was no reduction in the DNA signal of E2 and E6, demonstrating that the viral genome levels were not diminished in the cells (lanes 1–6, Fig. 2K). Following digestion of the DNA with TV exonuclease, mitochondrial DNA (which is circular, therefore episomal) was used as a control for episomal integrity and set as 100%. The signal generated by E2 primers demonstrates the E2 gene is on viral episomes (Fig. 2K, lane 9); it is slightly over 100% as the mitochondrial DNA samples had, on average, a small amount of degradation in the DNA samples. E6 has less integrity but remains over 50% episomal (Fig. 2K, lane 10), indicating that there is likely a mix of episomal and integrated genomes in the samples, with the integrated samples having lost the E2 gene. Following SMARCAL1 knockdown, the E2 and E6 signals become mostly integrated, as the signals detected are similar to those of GAPDH (Fig. 2K, lanes 13 and 14, and 17 and 18) and are significantly less than the levels detected with the control shRNA (lanes 9 and 10). It can be concluded from these studies that SMARCAL1 expression is essential for the maintenance of episomal viral genomes and is therefore essential for the HPV16 life cycle, and that the viral DNA becomes integrated following SMARCAL1 knockdown.

### SMARCAL1 is hyper-recruited to host replication forks in HPV16+ keratinocytes

**Fig 3:**
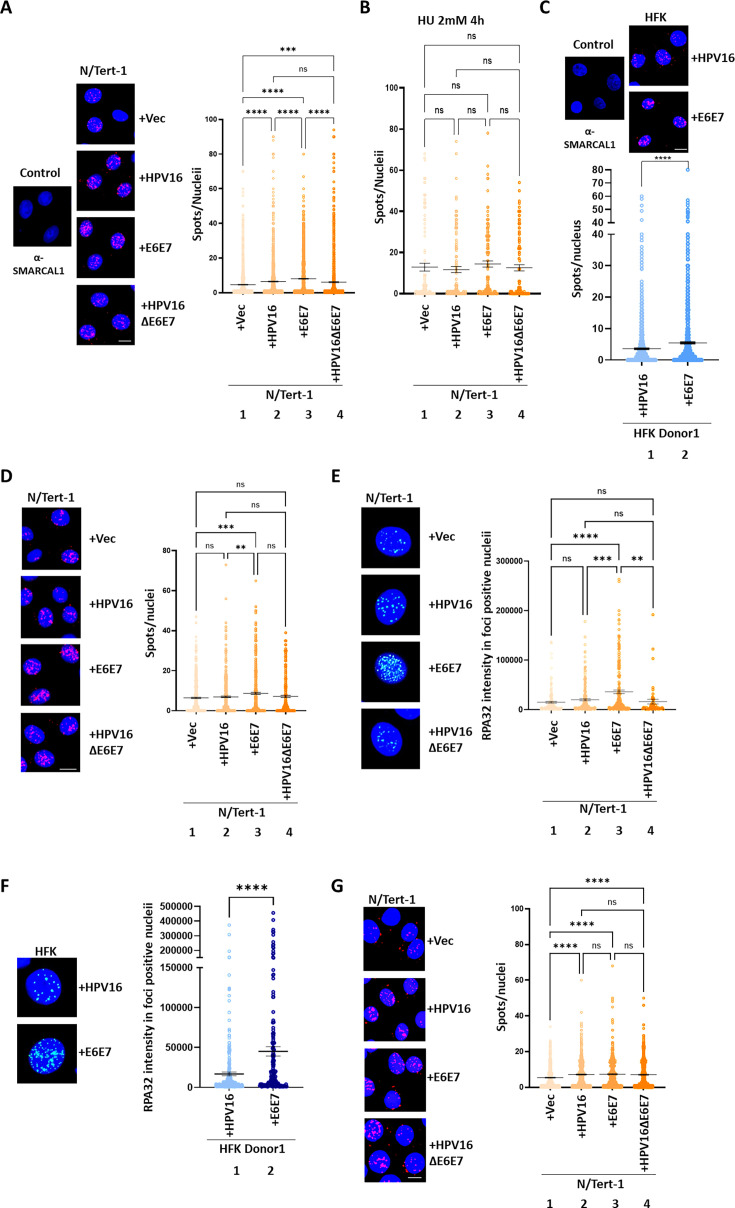
(**A**) SIRF assays were performed in N/Tert-1 cell lines to assess SMARCAL1 association with nascent DNA. Representative images are shown (left), with quantification of SMARCAL1 foci per nucleus shown on the right. Control samples include N/Tert-1+Vec cells stained with SMARCAL1 antibody alone. Scale bar, 20 µm. Statistical significance was determined by one-way ANOVA; ****P < 0.0001; ns, not significant. (**B**) SMARCAL1 recruitment to host replication forks in N/Tert-1 cells following treatment with 2 mM HU for 4 h was assessed by SIRF. Quantification revealed no significant differences among conditions (one-way ANOVA). (**C**) SIRF assays were repeated in HFK+HPV16 and HFK+E6E7 cell lines. Representative images are shown (left) with quantification on the right. Statistical significance was determined using the Mann-Whitney U-test; ****P < 0.0001. (**D**) SIRF biotin-biotin control assays were performed in the indicated cell lines. Representative images (left) and quantification (right) are shown. Statistical significance was assessed by one-way ANOVA; **P < 0.01, ***P < 0.001; ns, not significant. (**E**) RPA32-foci staining was analyzed by immunofluorescence (IF). The graphs show the intensity of RPA32-foci-positive N/Tert-1 cells, measured from two independent experiments (at least 50 nuclei). Representative images are presented on the left. Scale bar, 20 μm. Quantitation is shown on the right. The levels of statistical significance are indicated as *P*-values of <0.01 (**), <0.001 (***), and <0.0001 (****); ns, not significant (one-way ANOVA test). (**F**) RPA32-foci staining was analyzed by IF. The graphs show the intensity of RPA32-foci-positive HFK-E6E7 and HEK+HPV16 cells, measured from two independent experiments (at least 50 nuclei). Representative images are presented on the left. Scale bar, 20 μm. Quantitation is shown on the right. The levels of statistical significance are indicated as *P*-values of <0.01 (**), <0.001 (***), and <0.0001 (****); ns, not significant (Mann-Whitney U-test). (**G**) PLA assay was carried out using antibodies against the SMARCAL1 and RPA32 proteins. Representative images are presented on the left. Scale bar, 20 μm. Quantitation is shown on the right. The levels of statistical significance are indicated as *P*-values of <0.0001 (****); ns, not significant (one-way ANOVA test).

The essential nature of SMARCAL1 for the growth of HFK+HPV16 cells (Fig. 2) prompted us to analyze the recruitment of SMARCAL1 to host replication forks. To investigate this, we performed SIRF assays (i*n situ* analysis of protein *i*nteractions at DNA *r*eplication *f*orks). SIRF combines proximity ligation assays (PLA) with EdU click chemistry to detect protein co-localization with nascent DNA at a single-cell level (38). To allow direct comparison within isogenic cell lines, we used our previously characterized N/Tert-1+Vec (vector control), N/Tert-1+HPV16 (containing the entire HPV16 genome), N/Tert-1+E6E7 (expressing only the E6 and E7 oncogenes), and N/Tert-1+HPV16 ΔE6/E7, in which stop codons were introduced in the E6 and E7 oncogenes; HPV16 ΔE6/E7 serves as a control for the role of episomal replication without any effect of viral oncogene expression (35, 39, 40). The presence of HPV16 increased SMARCAL1 association with host replication forks. Figure 3A demonstrates that there is an increased presence of SMARCAL1 at host replication forks in N/Tert-1+HPV16 cells when compared with N/Tert-1+Vec, as demonstrated by the increased number of red dots observed (representative images to the left, quantitation to the right; compare lanes 1 and 2). SMARCAL1 was recruited to host replication forks in N/Tert-1+E6E7 cells at significantly higher levels when compared with N/Tert-1+HPV16 (Fig. 3A, compare lanes 2 and 3). SMARCAL1 was also more recruited to host replication forks in N/Tert-1+HPV16 ΔE6/E7 cells when compared with N/Tert-1+Vec control cells (Fig. 3A, compare lanes 1 and 4). This suggests that both viral replication *per se* and the viral oncogenes can regulate SMARCAL1 recruitment to host replication forks. Interestingly, the SMARCAL1 recruitment observed in N/Tert-1+HPV16 and N/Tert-1+HPV16 ΔE6/E7 cells is not significantly different (compare lanes 2 and 4), suggesting that when the entire viral genome is present, the regulation of SMARCAL1 recruitment is controlled by viral replication rather than the viral oncogenes. To confirm that all cells retained the capacity to hyper-recruit SMARCAL1 in the presence of replication stress, all cells were treated with 2 mM hydroxyurea (HU) for 4 h. Figure 3B demonstrates no difference in SMARCAL1 interaction with replication forks following HU treatment, demonstrating equivalent capacity for replication stress in all cell lines. The increased recruitment of SMARCAL1 by the viral oncogenes alone was reproducible in HFK+HPV16 (Fig. 3C, compare lanes 1 and 2 in the lower panel). To confirm that the increased recruitment of SMARCAL1 to host replication forks was not due to a simple increase in replication rates, we carried out PLA evaluating EdU incorporation by combining two anti-biotin antibodies (anti-mouse and anti-rabbit) (Fig. 3D). There was no difference in signal detected in any of the cell lines except for N/Tert-1+E6E7, where the signal was increased (compare lane 3 with all others). This suggests that the N/Tert-1+E6E7 cells grow more quickly, and that this contributes to the greater recruitment of SMARCAL1 in these cells (Fig. 3A). These results are confirmatory that, in cells containing replicating HPV16 genomes, the increased recruitment of SMARCAL1 to host replication forks is not due to a simple increase in EdU incorporation. RPA32 staining was elevated in all HPV16-containing N/Tert-1 cells but was highest in the E6/E7-only cells (Fig. 3E). This was reproducible in HFK immortalized by the viral oncogenes only or by the full genome (Fig. 3F). This indicates enhanced replication stress in the E6/E7 cells when compared with lines containing the full HPV16 genome.

To investigate whether increased SMARCAL1 interaction with chromatin is due to interaction with RPA, which is a marker of replication stress, we carried out PLA assays with SMARCAL1 and RPA (Fig. 3G) (41). There is an increase in SMARCAL1-RPA interaction in N/Tert-1+HPV16, N/Tert-1+E6/E7, and N/Tert-1+HPV16ΔE6E7 when compared to N/Tert-1+Vec cells (compare lanes 2–4 with lane 1). However, there is no difference in the SMARCAL1-RPA interaction between N/Tert-1+HPV16 and N/Tert-1+E6E7; this suggests an additional mechanism for SMARCAL1 recruitment in E6E7-only cells outside of RPA interaction, which would explain the enhanced SMARCAL1 recruitment to host replication forks in E6E7-only cells.

Overall, these results demonstrate that both replication of the viral genome and the viral oncogenes by themselves recruit SMARCAL1 to host replication forks for fork reversal resolution and ongoing replication. Our prior work demonstrated that N/Tert-1+HPV16 ΔE6/E7 cells retained an active DDR, providing a potential mechanism for the recruitment of SMARCAL1 to host replication forks in these cells (40).

### Knockdown of SMARCAL1 increases DNA damage in HFK+HPV16 cells

**Fig 4:**
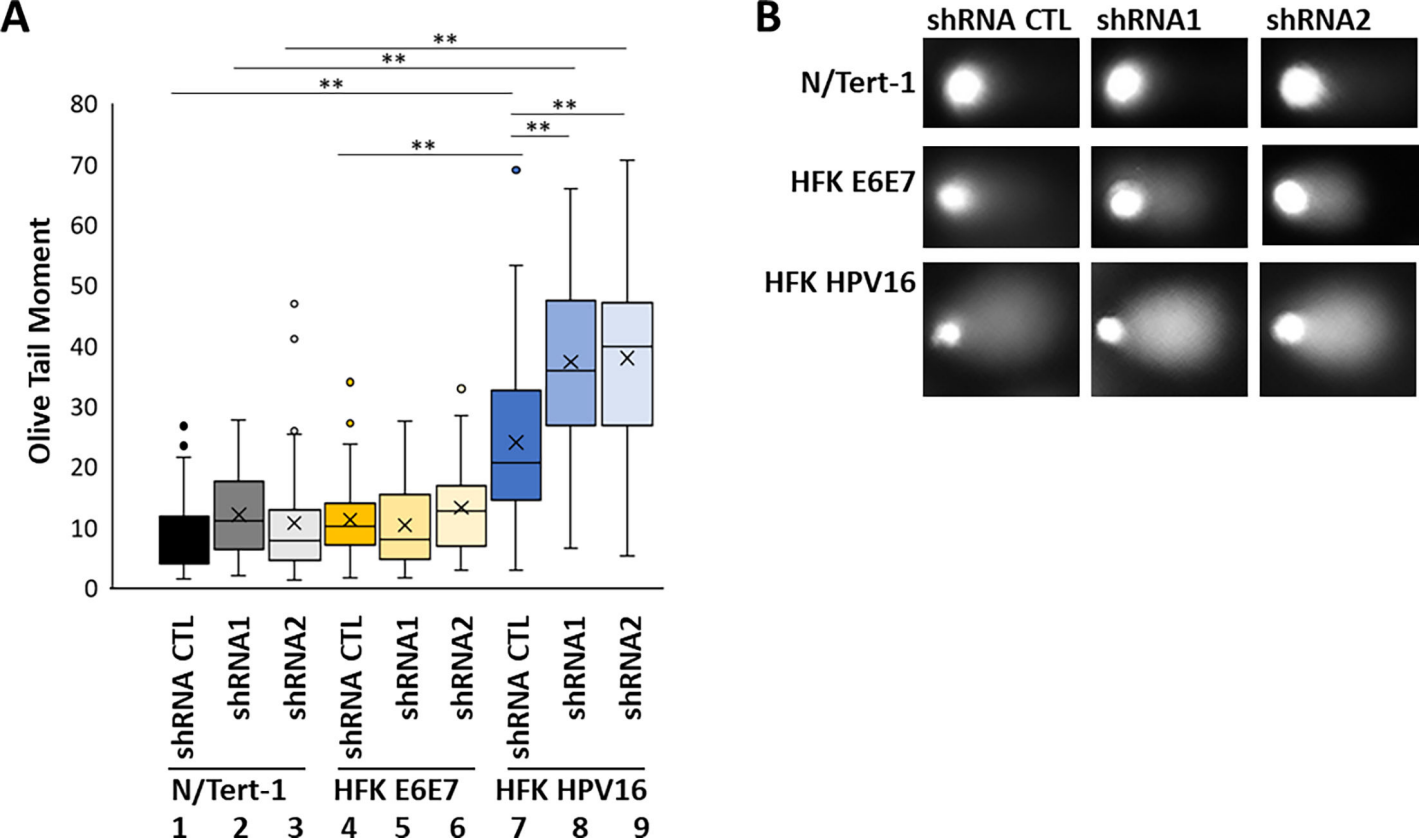
SMARCAL1 reduction induces DNA damage in HFK+HPV16 cells. (**A**) COMET assays were carried out with the indicated cell lines 48 h following treatment with 0.25 mM Dox. A summary of the olive tail moments is shown. (**B**) Representative DNA “tails” in the indicated cell types. **, *P*-value < 0.05.

The results so far have determined that SMARCAL1 is recruited to HPV16 E1-E2 replicating DNA (Fig. 1 and 2), that SMARCAL1 is essential for the growth of HFK+HPV16 cells (Fig. 2), and that SMARCAL1 is hyper-recruited to host replication forks in the presence of the HPV16 genome and the viral oncogenes (Fig. 3). The reason for the essential nature of SMARCAL1 in the growth of HFK+HPV16 was investigated. Figure 4A quantitates a COMET assay in N/Tert-1+Vec, HFK+HPV16, and HFK+E6E7 cells with and without SMARCAL1 expression. In shRNA control (shRNA CTL) cells, HFK+HPV16 have significantly higher olive tail moments (OTMs) when compared with N/Tert-1+Vec and HFK+E6E7 (compare lane 7 with lanes 1 and 4, respectively), indicative of increased DNA damage. When SMARCAL1 is knocked down by shRNA1 and 2 in HFK+HPV16 cells there is a significant increase in DNA damage when compared with HFK+HPV16 shRNA CTL (compare lanes 8 and 9 with lane 7). This increased DNA damage correlates with the critical nature of SMARCAL1 for HFK+HPV16 cell growth (Fig. 2). There is not a consistently significant increase in OTM in either N/Tert-1+Vec or HFK+E6E7 following SMARCAL1 knockdown (compare lanes 2 and 3 with lane 1, and lanes 5 and 6 with lane 4, respectively). As SMARCAL1 knockdown cannot consistently abrogate the growth of either of these cell lines, this further supports the idea that in HFK+HPV16 cells, SMARCAL1 knockdown results in growth attenuation due to increased DNA damage.

**Fig 5:**
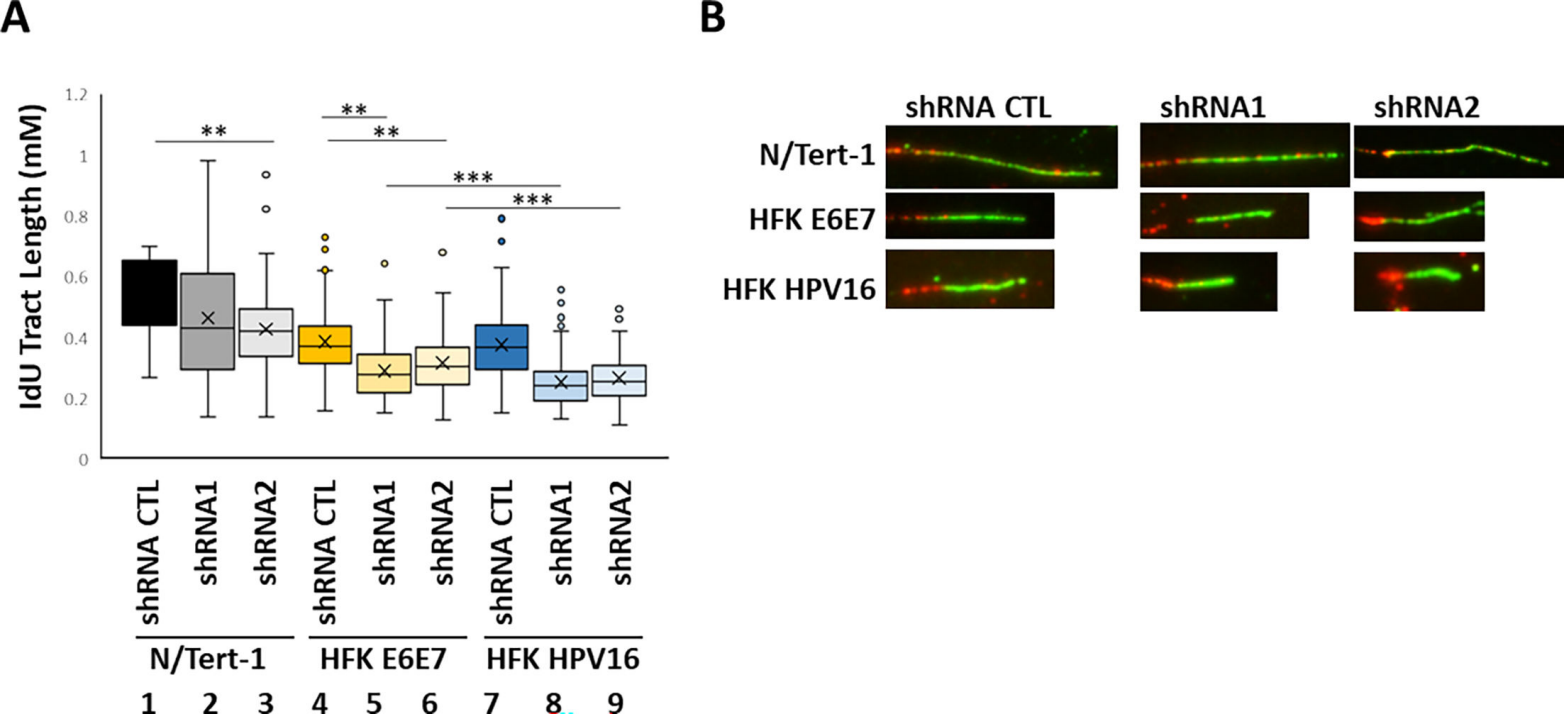
SMARCAL1 reduction attenuates host fork replication speeds in HFK+HPV16 cells. (**A**) Fiber assays were carried out with the indicated cell lines 48 h following treatment with 0.25 mM Dox. A summary of the “green” fiber length is shown. (**B**) Representative fibers from the indicated cell types. Asterisks highlight differences between samples, and the larger the number of asterisks, the higher the significance. *, *P*-value < 0.05 in all cases.

To determine whether HFK+HPV16 increased DNA damage in the absence of SMARCAL1 was the result of host replication forks slowing down (ultimately resulting in double strand breaks), fiber assays were utilized (Fig. 5). The quantitated results are shown in Fig. 5A. In HFK+HPV16 cells, SMARCAL1 knockdown significantly slowed replication fork speed (compare lane 7 with lanes 8 and 9), and the slowdown in fork speed was significantly greater than that observed in HFK+E6E7 cells (lanes 5 and 6) or in N/Tert-1+Vec cells (lanes 2 and 3). SMARCAL1 knockdown decreased fork speed in HFK+E6E7 cells (compare lanes 5 and 6 with lane 4), and was variable in N/Tert-1 cells (compare lanes 2 and 3 with lane 1). Overall, the results demonstrate that SMARCAL1 knockdown had the greatest effect on fork speed in HFK+HPV16 cells, and this correlates with the increased double-strand DNA breaks detected in these cells (Fig. 4).

## DISCUSSION

It is well established that HPV life cycles require activation of the DDR during epithelial differentiation (9). The reason for this activation is presumably to facilitate viral replication, which occurs more than once per cell cycle at early and late stages of the viral life cycle, with the potential to generate replication stress on the viral genome. Activation of the DDR promotes activation of several homologous recombination (HR) factors that would promote viral genome replication. For example, our previous work demonstrated that the host protein WRN is critical for E1-E2 DNA replication and for the viral life cycle (17). WRN is actively involved in several DNA repair pathways, including the resolution of replication fork reversals that can occur under replication stress conditions (42, 43). Given the critical role of WRN in the viral life cycle and its known involvement in stalled replication fork reversal resolution, we investigated the role of another protein involved in replication fork reversal resolution, SMARCAL1, in the viral life cycle (24, 25, 27, 28). The results presented here demonstrate that SMARCAL1 is recruited to E1-E2 replicating DNA and is required for maintaining high-fidelity E1-E2 replication. Importantly, depletion of SMARCAL1 results in viral genome integration; therefore, SMARCAL1 is essential for the HPV16 life cycle as it is required for viral genome maintenance. Our understanding of mechanisms and factors involved in maintaining an episomal viral genome is incomplete, and the results presented here demonstrate SMARCAL1 is critical for viral genome maintenance. Downregulation of SMARCAL1 reduces expression of E1 and E2, and this may play a critical role in mediating SMARCAL1’s effect on the HPV16 life cycle (Fig. 1). Therefore, SMARCAL1 can be added to the growing list of DNA damage/repair factors that are important during the viral life cycle. An interesting aspect of our results is that elimination of SMARCAL1 from cells preferentially attenuates the growth of keratinocytes containing the entire HPV16 genome. Many studies have demonstrated host factors are important for viral genome maintenance, as their knockdown promotes viral genome integration, including SIRT1 and members of the MRN complex, but that the presence of these factors is not essential for the growth of cells with entire HPV genomes (19, 44–46). The essential nature of SMARCAL1 for HPV16 positive cell growth (but not those immortalized by the viral oncogenes E6 and E7) prompted us to investigate the mechanism of why SMARCAL1 depletion preferentially abrogates growth in cells containing the full HPV16 genomes.

Using SIRF assays, we demonstrate that SMARCAL1 is recruited to host DNA replication forks in cells containing the entire HPV16 genome, as well as in cells expressing the viral oncogenes E6 and E7 only. Additionally, a viral genome with stop codons in E6 and E7 recruits SMARCAL1 to host replication forks to the same extent as cells containing the full genome. The viral oncogenes by themselves are “better” promoters of SMARCAL1 localization to host replication forks. To reconcile these observations, we propose that HPV16 induces SMARCAL1 recruitment through at least two mechanisms: In cells containing the viral oncogenes alone, the replication stress induced (demonstrated by the elevated presence of RPA at host replication forks) promotes recruitment of SMARCAL1 to stalled host forks. By contrast, in cells containing the full HPV16 genome, replication stress, as measured by RPA recruitment, is not increased, yet SMARCAL1 shows enhanced recruitment to host replication forks. Recently, we demonstrated that the presence of E2 (present in the HPV16 cells, not present in the oncogene-only cells) induces an active DDR during mitosis (we have extended these studies to demonstrate E2 activates the DDR during differentiation, not shown) (31). Consistent with this, cells containing the full HPV16 genome exhibit increased DNA damage, as reflected by increased COMET tails detected in cells containing HPV16 versus the E6 and E7 oncogenes only. We propose that this E2-driven DNA damage may alter the requirements at host replication forks during S phase, promoting recruitment of SMARCAL1, which is subsequently required to limit further accumulation of DNA damage. This model also explains the increased SMARCAL1 recruitment to host replication forks in HPV16 ΔE6/E7 cells. Therefore, there are two different mechanisms the virus uses to induce SMARCAL1 to host replication forks, one related to viral replication (perhaps E2 expression) and one related to oncogene-induced replication stress. The reason that the two different mechanisms are not additive in cells with the entire HPV16 genome could be that the viral oncogenes are not as “active” in cells containing the entire genome, as traditional E6 and E7 targets, p53 and pRB respectively, are expressed in cells containing the entire HPV16 genome, but not in cells immortalized only with the viral oncogenes (47). We are currently investigating the reasons for the differences in oncogene function under different circumstances and further elucidating the mechanism of SMARCAL1 recruitment to host DNA replication forks by HPV16.

SMARCAL1 is also critical for viral replication as it is recruited to E1-E2 replicating DNA, and depletion of SMARCAL1 results in a reduction in E1-E2 replication fidelity. How SMARCAL1 is recruited to the viral replication forks is unclear; it could be related directly to the DNA structures generated on the viral genome. The E2-SMARCAL1 interaction is disrupted in the presence of E1, suggesting that E1 and SMARCAL1 interact with E2 in similar areas. Interestingly, WRN interacts with E1 in the domain of E1 that interacts with E2 (17). This suggests an intricate interaction of the viral replication factors with host DDR proteins that is required for viral replication and execution of the viral life cycle.

Overall, the results demonstrate a critical role for SMARCAL1 in the viral life cycle. The results also present a novel approach to the targeting of HPV16-infected cells: inhibition of SMARCAL1 enzyme function. Unlike other DNA damage factors involved in the viral life cycle, whose knockout induces viral genome integration but does not affect cell growth, SMARCAL1 depletion attenuates the growth of HPV16 positive keratinocytes. Future studies will focus on gaining an in-depth understanding of why SMARCAL1 is essential for the growth of HPV16 positive cells and developing SMARCAL1 as an antiviral therapeutic target.

## MATERIALS AND METHODS

### Cell culture

C33a cells were obtained from ATCC (Manassas, VA, USA) and cultured in DMEM (Invitrogen) supplemented with 10% fetal bovine serum. Human foreskin keratinocytes (HFKs) were immortalized with HPV16 as described previously (48) or with 16 E6E7 by retroviral delivery using pLXSN16E6E7, a gift from Denise Galloway (Addgene plasmid #52394). HFKs were cultured in DermaLife-K Complete media (LifeLine Cell Technologies). Mitomycin C-treated 3T3-J2 fibroblast feeders were plated 24 h prior to seeding N/Tert-1 or HFK cells on top of the feeders, in their respective cell culture media. Media were refreshed and 3T3-J2s supplemented as required. N/Tert-1 cells were cultured in keratinocyte serum-free medium (K-SFM) (Invitrogen) supplemented with bovine pituitary extract, EGF (Invitrogen), 0.3 mM calcium chloride (MilliporeSigma; 21115), and 7.5 µM hygromycin. In all cases, cells were incubated at 37°C in a 5% CO_2_/95% air atmosphere, routinely passaged before reaching confluency, and screened for mycoplasma.

### Cell synchronization

HFK cells immortalized with HPV16 genomes (HFK+HPV16) were cultured with Mitomycin C-treated 3T3-J2 fibroblasts. Cells were plated at 5 × 10^5^ density onto 100 mm^2^ plates. The cells were treated with 2 mM thymidine diluted in their respective medium for 16 h. Cells were then washed two times with PBS and recovered in their respective medium. After 8 h, to block the cells at the G1/S phase, a second dose of 2 mM thymidine was added, and the cells were incubated for 17 h. The cells were then washed two times with PBS and recovered at 0 h (G1/S phase) and 19 h (M1 phase).

### Generation of inducible SMARCAL1 knockdown cell lines

A set of three SMARTvector human Inducible Lentiviral shRNA plasmids targeting SMARCAL1 were purchased from Horizon, along with one empty vector control. C33a and keratinocytes immortalized with hTERT (N-Tert/1), E6E7 (HFK-E6E7), or HPV16 (HFK-HPV16) were infected with the resulting lentiviruses and selected with puromycin to generate lines expressing inducible control (CTL) or SMARCAL1 shRNA (shRNA1–3). Optimal doxycycline treatment for shRNA induction was determined by treatment of cells followed by PCR and western blot analysis; 0.25 µM doxycycline for 48 h was found to induce shRNA without toxicity.

### Colony formation assay

Cells were seeded into 6-well plates at a density of 1 × 10⁴ cells per well and cultured in the presence of vehicle or 5 µM doxycycline to induce shRNA expression. Cells were maintained until colonies were visible in vehicle-treated wells (approximately 7 days). Media was removed, and wells were washed twice with PBS before staining with 0.5% crystal violet (1 mL per well) for 30 min at room temperature (RT) with gentle agitation. Wells were washed five times with PBS, air-dried, and imaged using an Odyssey CLx Imaging System. Colony area was quantified using ImageJ.

### ChIP

Cross-linking and chromatin extraction of C33a cells were carried out as previously described (13). For chromatin extraction from keratinocytes, the ChIP-It Enzymatic kit (Active Motif) was utilized, as the protocol dictated, including dounce homogenization to disrupt cells and incubation with shearing enzymes for 7.5 min with frequent agitation. In both cases, sheared chromatin was incubated with 1 µg primary antibody (HA, Abcam ab9110; SMARCAL1 [Abcam ab154226]; E2 Sheep) and protein-A-conjugated magnetic beads, rotating overnight at 4°C. Beads were then washed, and DNA was eluted via proteinase K digestion. Precipitated viral DNA was measured via qPCR using the following primers: HPV16 LCR F: 5′-GAAAACGAAAAGCTACACCCA-3′, R: 5′-CAATGAATAACCACAACACAATTA-3′; HPV16 E2 F: 5′- TGGAAGTGCAGTTTGATGGA-3′, R: 5′-CCGCATGAACTTCCCATACT-3′; HPV16 E5 F: 5′-CACAACATTACTGGCGTGCT-3′, R: 5′- ACCTAAACGCAGAGGCTGCT-3′; HPV16 E6 F: 5′-AATGTTTCAGGACCCACAGG -3′, R: 5′-GCATAAATCCCGAAAAGCAA-3′. Percentage pulldown was calculated by normalizing *C*_*t*_ to adjusted input: ∆*C*_*t*_ = *C*_*t*_ [IP] – (*C*_*t*_ [input] – log_2_10), followed by % input = 2^–∆*Ct*^ × 100% to input: %.

### Transient replication assay

C33a cells were plated out at 5 × 10^5^ in 10 cm dishes. The following day, plasmid DNA was transfected using the calcium phosphate method. Three days post-transfection, low molecular weight DNA was extracted using the Hirt method as previously described (49). The digested sample was extracted twice with phenol:chloroform:isoamyl alcohol (25:24:1) and precipitated with ethanol. Following centrifugation, the DNA pellet was washed with 70% ethanol, dried, and resuspended in a total of 150 µL water. Forty-two microliters of sample were digested with DpnI (New England Biolabs, Ipswich, MA, USA) overnight to remove unreplicated pOri16LacZ; the sample was then digested with ExoIII (New England Biolabs) for 1 h. Replication was determined by real-time PCR, as described previously (32).

### qRT-PCR

Total RNA was isolated using the SV Total RNA Isolation System (Promega) according to the manufacturer’s instructions. Two micrograms of RNA were reverse transcribed using the High-Capacity cDNA Reverse Transcription Kit (Applied Biosystems). qPCR was performed using PowerUp SYBR Green Master Mix (Applied Biosystems) on a 7500 Fast Real-Time PCR System as previously described (13). Gene expression was quantified relative to GAPDH using the 2^−ΔΔCT^ method. Primer sequences were as follows: GAPDH F: 5′-GGAGCGAGATCCCTCCAAAAT-3′, R: 5′-GGCTGTTGTCATACTTCTCATGG-3′; SMARCAL1 F: 5′-CACCAAGGACAAAACTAAACAGCAGCAG-3′, R: 5′-GAGGTGGAGCCATCGATGCGGA-3′, E2 F: 5′-TGGAAGTGCAGTTTGATGGA-3′ E2 R: 5′- CCGCATGAACTTCCCATACT-3′, E6 F: 5′-GAGAACTGCAATGTTTCAGGACC-3′, R: 5′-TGTATAGTTGTTTGCAGCTCTGTGC-3′, E7 F: 5′-GTGTGACTCTACGCTTCGGT-3′, R: 5′-AGAACAGATGGGGCACACAA-3′.

### DNA mutagenesis assay

Replication fidelity was determined by blue-white assay as described previously (33). Essentially, DNA from the transient replication assay was resuspended in 150 µL of 10% glycerol; 75 µL was electroporated into DH10B bacteria and plated onto kanamycin lysogeny broth agar containing 100 µg/mL X-gal. Following incubation overnight at 37°C, bacterial colonies were observed and counted; blue colonies correspond to those with no mutations in the LacZ gene, downstream of pOri, and the white colonies correspond to those containing mutations. Thus, the ratio of blue colonies to white provides a readout of replication mutation frequency.

### *In situ* analysis of protein interactions at DNA replication forks (SIRF)

Exponentially growing cells were seeded onto microscope chamber slides. The day of the experiment, cells were incubated with 125 µM 5-ethynyl-2′-deoxyuridine (EdU) for 8 min. Cells were pre-extracted in 0.5% Triton X-100 for 10 min on ice and fixed with 2% paraformaldehyde (PFA) in 1× PBS for 15 min at room temperature. Cells were then permeabilized in 0.25% Triton X-100 for 15 min at RT. To detect EdU, the Click-iT EdU Alexa Fluor Imaging Kit (Invitrogen) using 5 µM biotin-azide was used for 30 min at room temperature. Cells were washed with 1× PBS for 5 min and blocked in 3% bovine serum albumin (BSA)/PBS for 20 min. The primary antibodies used were as follows: SMARCAL1 (Abcam, 1:400), mouse anti-biotin (Jackson, 1:1,200). The negative controls were obtained by using only one primary antibody. Samples were incubated with secondary antibodies conjugated with PLA (proximity ligation assay) probes MINUS and PLUS: the PLA probe anti-mouse PLUS and anti-rabbit MINUS (or NaveniFlex equivalent). The incubation with all antibodies was carried out in a humidified chamber for 1 h at 37°C. Next, the PLA probes MINUS and PLUS were ligated using two connecting oligonucleotides to produce a template for rolling-cycle amplification. After amplification, the products were hybridized with red fluorescence-labeled oligonucleotide. Samples were mounted in ProLong Gold anti-fade reagent with 4,6-diamidino-2-phenylindole (DAPI) (blue). Images were acquired randomly using an Eclipse 80i Nikon Fluorescence Microscope, equipped with a virtual confocal system. The analysis was carried out by counting the PLA spots for each nucleus and was plotted and analyzed using GraphPad Prism.

### PLA

PLA was performed using the Duolink *In Situ* Red Starter Kit (Sigma) following the manufacturer’s protocol. Fixed cells were incubated with primary antibodies against 53BP1 (Invitrogen, PA1-16565) and γH2AX (Cell Signaling Technology, 9718), SMARCAL1 (1:1,000, Abcam, ab154226), RPA34-20 (1:1,000, (Merck). Ligation and amplification were performed at 37°C. PLA foci were visualized by fluorescence microscopy and quantified using ImageJ.

### IF

Cells were grown on coverslips in 35 mm dishes until 70%–80% confluence. Subsequently, cells were washed with ice-cold PBS for 5 min, pre-extracted on ice with 0.5% Triton X-100 for 10 min, and fixed with 3% PFA/2% sucrose at RT for 10 min. After two washes in PBS for 5 min, 3% BSA was added for 15 min at RT. Then, cells were incubated with anti-RPA34-20 (Merck, 1:400) diluted in a 1% BSA/0.1% saponin in PBS solution, for 1 h at 37°C in a humidifier chamber. After three washes in PBS for 5 min, secondary antibody (Alexa Fluor 488-conjugated goat anti-mouse IgG (H + L), highly cross-adsorbed; Life Technologies) was added at 1:200 dilution in a 1% BSA/0.1% saponin in PBS solution, for 1 h at RT. Counterstaining was performed with 0.5 μg/mL DAPI. Slides were analyzed with an Eclipse 80i Nikon Fluorescence Microscope, equipped with a Video Confocal system at 40× magnification. Fluorescence intensity for each sample was then analyzed using ImageJ software.

### Single-cell gel electrophoresis/comet assay

DNA breakage induction was evaluated by comet assay (single-cell gel electrophoresis) in alkaline conditions. Ten thousand cells were harvested by trypsinization and resuspended in 100 µL of 1% (wt/vol) Seaplaque agarose (Lonza)/PBS. The cell-agarose mix was then transferred onto slides pre-treated with 1% agarose/PBS and left to set at 4°C in the dark. Slides were immersed in freshly prepared cold lysing solution (2.5 M NaCl, 83 mM sodium-EDTA, 10 mM Tris, 1% Triton X-100, 10% DMSO, pH10) and incubated for 1.5 h at 4°C in the dark. Following this incubation, the slides were removed from the lysing solution and placed in a horizontal gel electrophoresis tank, side-by-side, with the agarose gels in the same plane of orientation in line with the anode. The electrophoresis tank was then filled with fresh electrophoresis buffer (0.3 M NaOH, 1 mM EDTA), and the slides were incubated in this buffer for 20 min. Cells were electrophoresed for 30 min at 20 V. Following electrophoresis, slides were washed with neutralization buffer (100 mM Tris, pH 7.4) three times at 4°C and stained for 3 min with 50 μL of 10 μg/mL ethidium bromide in distilled water. Excess stain was removed by flooding the slides with PBS, and coverslips were applied prior to viewing using a fluorescence microscope (Keyence). DNA damage was quantified using CaspLab (kkoncr), 100 cells were analyzed per sample, 50 cells per duplicate slide. The parameter used to reflect the amount of DNA damage was the Olive Relative Tail Moment (OTM), which is proportional to the % DNA in the tail multiplied by the distance between the two centers of mass in the head and tail (50). This parameter was decided upon as the most appropriate descriptor of the fraction of DNA released from the cell nucleus and the distance migrated by this DNA in an electric field.

### DNA fiber analysis

Replication fork speed was measured using the DNA fiber assay. DNA fibers were prepared and analyzed as described (51, 52). First, cells were treated with 25 μM IdU for 20 min, and then cells were washed twice with PBS and incubated for 20 min in fresh medium containing 100 μM CldU. Cells were harvested by trypsinization and pellets resuspended in ice-cold PBS. Two to 4 μL of ice-cold cell suspension was transferred to the top of each microscope slide, allowed to dry for 30 s before adding 12 μL fresh lysis buffer (200 mM Tris-HCl, pH 7.5; 50 mM EDTA; 0.5% SDS) and stirring with the pipette tip. Slides were tilted to an angle of 25° to 40° for 2 min and laid flat to air dry completely in the dark (approximately 5–15 min). Slides were fixed in fresh ethanol/acetic acid 3:1 for 15 min. Once dried, these were subjected to immunodetection of labeled tracks, using the following primary antibodies were used: rat anti-CldU/BrdU (Abcam) and mouse anti-IdU/BrdU (Becton Dickinson). Images were acquired randomly from fields with untangled fibers using a Keyence BZ-X800 microscope. The length of labeled tracks was measured using the ImageJ software. A minimum of 100 individual fibers was analyzed for each experiment, and each experiment was repeated two times.

### Immunoblotting

Specified cells were trypsinized, washed with PBS, and resuspended in 2× pellet volume NP-40 protein lysis buffer (0.5% Nonidet P-40, 50 mM Tris [pH 7.8], 150 mM NaCl) supplemented with protease inhibitor (Roche Molecular Biochemicals) and phosphatase inhibitor cocktail (MilliporeSigma). Cell suspension was incubated on ice for 20 min and then centrifuged for 20 min at 184,000 rcf at 4°C. Protein concentration was determined using the Bio-Rad protein estimation assay according to manufacturer’s instructions. 50 μg protein was mixed with 2× Laemmli sample buffer (Bio-Rad) and heated at 95°C for 5 min. Protein samples were separated on Novex 4–12% Tris-glycine gel (Invitrogen) and transferred onto a nitrocellulose membrane (Bio-Rad) at 35V overnight using the wet-blot transfer method. Membranes were then blocked with Odyssey (PBS) blocking buffer diluted 1:1 with PBS and probed with indicated primary antibody diluted in Odyssey blocking buffer. Membranes were washed with PBS supplemented with 0.1% Tween (PBS-Tween) and probed with the Odyssey secondary antibody (goat anti-mouse IRDye 800CW or goat anti-rabbit IRDye 680CW) (Li-Cor) diluted in Odyssey blocking buffer at 1:10,000. Membranes were washed twice with PBS-Tween and an additional wash with 1× PBS. After the washes, the membrane was imaged using the Odyssey CLx Imaging System and ImageJ was used for quantification, utilizing GAPDH as an internal loading control. Primary antibodies used for western blotting studies are as follows: 16E2 monoclonal B9 1/500 (31), GAPDH 1/10,000 (Santa Cruz, sc-47724), HA-Tag (Abcam, ab9110), SMARCAL1 1/1,000 (Abcam, ab154226), and cyclin B 1/1,000 (Cell Signaling Technology, 4138).

### IP

Cell lysate was prepared as described above. 500 µg of the lysate was incubated with lysis buffer (0.5% Nonidet P-40, 50 mM Tris [pH 7.8], and 150 mM NaCl), supplemented with protease inhibitor (Roche Molecular Biochemicals) and phosphatase inhibitor cocktail (MilliporeSigma) to a total volume of 500 µL. A primary antibody of interest or a FLAG-tag antibody (used as a negative control) was added to this prepared lysate and rotated at 4°C overnight. The following day, 40 µL of protein A beads per sample (MilliporeSigma; equilibrated to lysis buffer as mentioned in the manufacturer’s protocol) was added to the above mixture and rotated for another 4 h at 4°C. The samples were gently washed with 500 µL lysis buffer by centrifugation at 1,000 × *g* for 2–3 min. This wash was repeated three times. The bead pellet was resuspended in 4× Laemmli sample buffer (Bio-Rad), heat-denatured, and centrifuged at 1,000 × *g* for 2–3 min. The supernatant was applied to an SDS-PAGE system to separate and resolve proteins and was then transferred onto a nitrocellulose membrane using wet-blot transfer method. The membrane was probed for the presence of the indicated proteins as mentioned in the description of western blotting above.

### Exonuclease 5 (T5) assay

PCR-based analysis of viral genome status was performed using established methods (53). Briefly, 20 ng genomic DNA was either treated with exonuclease V (RecBCD, NEB), in a total volume of 30 μL, or left untreated for 1 h at 37°C followed by heat inactivation at 95°C for 10 min. Two nanograms of digested/undigested DNA was then quantified by real-time PCR. Separate PCRs were performed to amplify HPV16 E6 F: 5′-TTGCTTTTCGGGATTTATGC-3′ R: 5′- CAGGACACAGTGGCTTTTGA-3′, HPV16 E2 F: 5′-TGGAAGTGCAGTTTGATGGA-3′ R: 5′-CCGCATGAACTTCCCATACT-3′, human mitochondrial DNA F: 5′-CAGGAGTAGGAGAGAGGGAGGTAAG-3′ R: 5′-TACCCATCATAATCGGAGGCTTTGG-3′, and human GAPDH DNA F: 5′-GGAGCGAGATCCCTCCAAAAT-3′ R: 5′- GGCTGTTGTCATACTTCTCATGG-3′. Episomal status was calculated by ΔCt(Exo-no Enzyme) and then comparison of dCt(HPV gene) to dCt(mitochondrial DNA) and dCt(GAPDH).

### Statistics

Standard error was calculated from three independent experiments and significance determined using a Student’s *t*-test or Mann-Whitney for non-parametric distributions. One-way ANOVA with Tukey’s test was used for multiple comparisons.
